# Characteristics and Effects of Multiple and Mixed Funding Flows to Public Healthcare Facilities on Financing Outcomes: A Case Study From Nigeria

**DOI:** 10.3389/fpubh.2019.00403

**Published:** 2020-01-15

**Authors:** Obinna Onwujekwe, Chinyere Mbachu, Uche Ezenwaka, Ifeyinwa Arize, Nkoli Ezumah

**Affiliations:** ^1^Health Policy Research Group, Department of Pharmacology and Therapeutics, College of Medicine, University of Nigeria Enugu Campus, Enugu, Nigeria; ^2^Department of Health Administration and Management, College of Medicine, University of Nigeria Enugu Campus, Enugu, Nigeria; ^3^Department of Community Medicine, College of Medicine, University of Nigeria Enugu Campus, Enugu, Nigeria; ^4^Institute of Public Health, College of Medicine, University of Nigeria Enugu Campus, Enugu, Nigeria

**Keywords:** multiple funding flows, public hospitals, provider behaviors, health financing, Nigeria

## Abstract

**Introduction:** Most public hospitals in Nigeria are usually financed by funding flows from different health financing mechanisms, which could potentially trigger different provider behaviors that can affect the health system goals of efficiency, equity, and quality of care. The study examined how healthcare providers respond to multiple funding flows and the implications of such flows for achieving equity, efficiency, and quality.

**Methods:** A cross-sectional qualitative study of selected healthcare providers and purchasers in Enugu state was used. Four public hospitals were selected—two tertiary and two secondary; because they received funding from more than one healthcare financing mechanism. Key informants were individual healthcare providers and decision-makers in the hospitals, State Ministry of Health, National Health Insurance Scheme and Health Maintenance Organizations. Service users from each hospital were purposively selected for focus group discussions (FGDs). A total of 66 key informant interviews and 8 FGDs were conducted.

**Findings:** The multiple flows that were received by public hospitals varied by type of health facility (Secondary vs. Tertiary), ownership of health facility (Federal government vs. State government) and population served. Out-of-pocket payment (OOP) and government budget were the only recurring forms of funding to all the public hospitals. It was found that multiple funding flows, generate different signals to service providers, resulting in positive and negative consequences. The results also showed that multiple flows lead to predictability and stability of funding to public hospitals. Hospital Managers and administrators reported that multiple flows increased their financial pool and capacity to undertake capital projects and enabled the provision of a wider range of services to clients. Multiple sources of funding also give a sense of security to health facilities, because there would always be a back-up source of funding if one flow delays or defaults in payment. Nevertheless, health providers were seen to shift resources from less attractive to more attractive flows in response to the relative size perceived adequacy, predictability, and flexibility of funding flow. Patients were also shifted from less predictable to more predictable funding flows and providers charged different rates to different funding flows to make up for the inadequacies in some sources of funding. The negative consequences of multiple funding flows on provider behavior that was reported in the study were wastage/under-utilization of resources, differential quality of care provided to clients, and inequities in resource distribution and access to health services. In some instances, providers' responses resulted in better quality of care for clients and improved access to services that were not ordinarily available or clients could not have been afforded.

**Conclusion:** Multiple funding flows to public hospitals are beneficial as well as constraining to health providers. They can be beneficial in ensuring that hospitals have a ready and predictable pool of funds to render services with. However, they could be detrimental to some patients that could be charged more for some services that other patients pay less and may also lead of provision of differential quality of services to different payments depending on the funding flows that are used to purchase services for them. Ultimately, some of the consequences of multiple funding flows if not properly managed, will affect health systems goals of equity, efficiency and quality of care, either positively or negatively.

## Introduction

Health system financing mechanisms are critical in ensuring Universal Health Coverage as they determine the availability, affordability, and acceptability of health services to the people (1). Health system financing functions include mobilizing revenues, pooling resources and purchasing services (1, 2). Evidence from all over the world suggests that equity and access are greater when healthcare is funded through taxation or social health insurance than when funded from private health insurance (PHI) or Out-Of-Pocket payments (3, 4). Purchasing is the health financing function through which pooled funds are transferred to health care providers (5).

Purchasing is a core function of health care financing that involves the transfer of pooled resources to healthcare providers in exchange for healthcare services (6) and it is high on the health financing agenda as it is critical in achieving universal health coverage (7). Purchasing entails that purchasers act as agents for the people in the purchase of healthcare services (7). To fulfill this role effectively, it is important for purchasers needs to ensure that there are effective mechanisms in place to determine people's needs, preferences, and values in purchasing decisions and to hold themselves accountable to the population for which they are responsible (7).

All the states in Nigeria rely on a mixture of government budget (from general tax revenue), health insurance (social and private), external funding (donor funding), and private out-of-pocket spending to finance health care (8). In Nigeria, public funding accounts for about 25% of total health spending while the private sector provides 75% of the funding, with household out-of-pocket expenditure accounting for 95% of the private sector expenditure (9). However, the sources of funding to healthcare service providers in Nigeria vary at different levels of health facilities and in different states.

The federal, state and local government areas provide budgets from general tax revenue to the Federal Ministry of Health (FMOH), State Ministries of Health (SMOH), and Local Government Areas' (LGA) health authorities (LGAHA), respectively, who then act as purchasing organizations using budget flows, respectively, at the three tiers of government to allocate budgets for providers at health facilities (7). The FMOH, SMoH, and LGAHA also define a minimum package of health care services which covers promotion, preventive, and curative care at primary and secondary care levels, and includes services for communicable and non-communicable diseases, child survival, safe motherhood, nutrition, health education, laboratory services and community mobilization (10). The FMOH, SMoH, and LGA pay salaries of public servants.

Another existing major health financing mechanism in Nigeria is the Formal sector health insurance Programme (FSSHIP), which is run by the National Health Insurance Scheme (NHIS). It is a mandatory scheme for employees in the formal sector (11). The NHIS contracts private, for-profit Health Maintenance Organizations (HMOs) to administer the purchasing system and channel resources to providers. Healthcare providers receive capitation payments for primary healthcare services and fee-for-service for secondary services (6). A mix of NHIS-accredited public and private health care providers are contracted to deliver services a standard benefit package. FSSHIP enrollees are allocated to specific HMOs, but can choose their primary health care providers from an NHIS accredited provider list (7).

**Figure 1:**
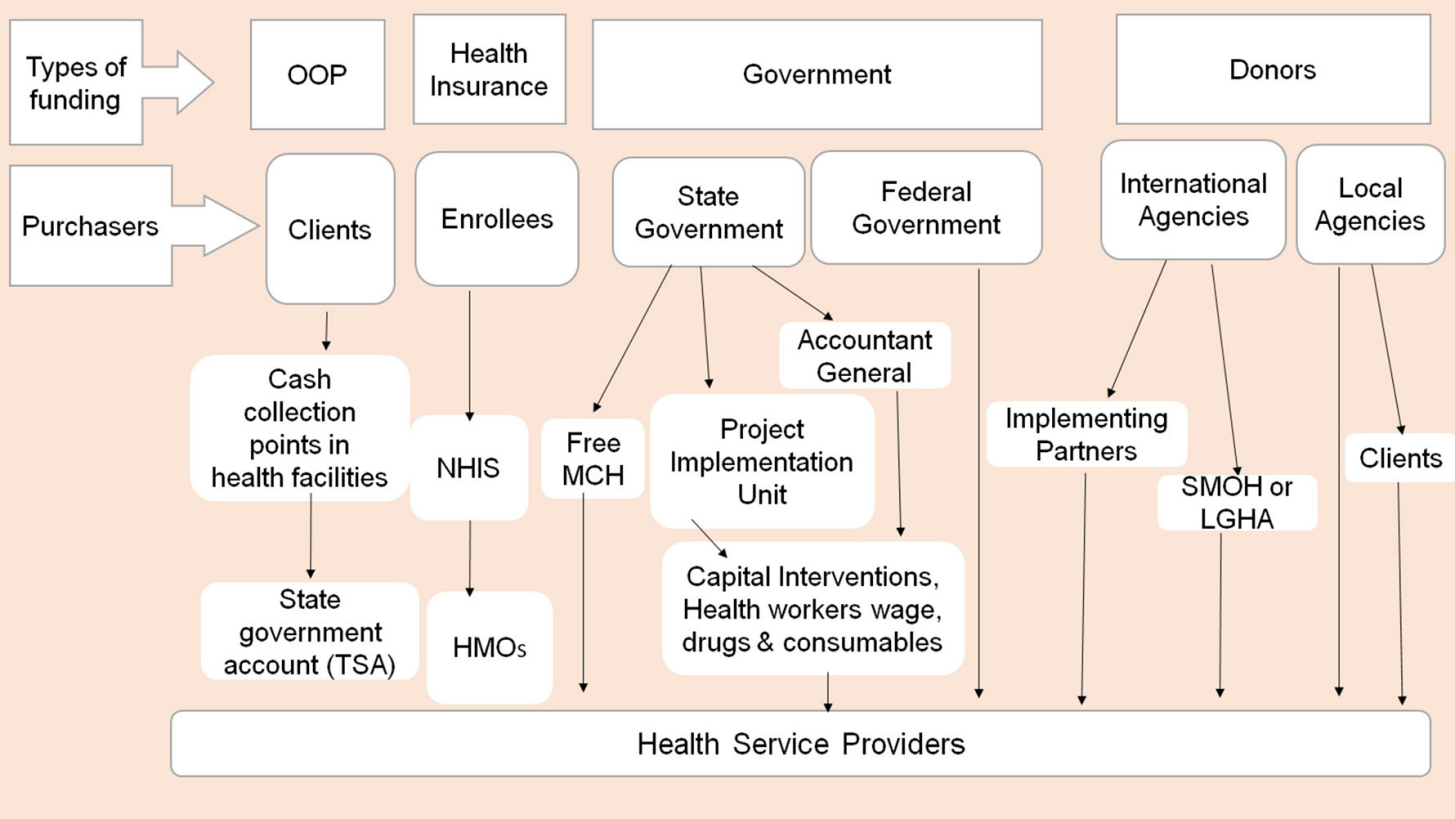
Different funding flows to health providers in Nigeria.

Figure 1 illustrates the multiple funding flows to healthcare providers in Nigeria. It shows that many healthcare providers in Nigeria receive funding flows from different health financing mechanisms. These include budgets, health insurance, out-of-pocket payments, donor funding, and others. A funding flow refers to any transfer of funds, in cash or kind, from a purchaser to a healthcare provider that is characterized by a distinct combination of arrangements (12). Each funding flow is characterized by different payment mechanism, provider payment rates, contractual agreement, reporting requirement, decision space, and accountability mechanisms.

Purchasing for health in Nigeria is undertaken by government at all levels through the Ministries of Health and LGAHA, the National Health Insurance Scheme (NHIS), the National Primary Healthcare Development Agency (NPHCDA), HMOs, PHI, Community based health insurance (CBHI), development partners, non-governmental organizations (NGO), and households. The purchasers transfer funds to healthcare providers for the provision of services.

Economic theory suggests that different payment mechanisms can produce different behaviors on the part of providers that can affect efficiency, equity and quality of care (13). The design and implementation of parallel funding flows are likely to impact on the financial resilience of healthcare providers and create signals to which providers respond in both intended and unintended ways. Parallel mechanisms, unless designed as part of an integrated system, can undermine the ability of purchasers to undertake strategic purchasing. The report of the RESYST multi-country study on strategic purchasing found that where multiple purchasing mechanisms operate within a health system, it is important to understand the signals sent by each mechanism and funding flow and determine how these together influence the behaviors of healthcare providers (5).

It is important to understand the signals sent by each mechanism and funding flow, and determine how these together influence the behavior of healthcare providers. The signals sent to providers by a mix of funding flows are likely to be shaped by their relative size, provider payment mechanisms, provider payment rates, the services purchased, the population covered, levels of supervision, accountability requirements, and interactions between all of these factors.

Parallel mechanisms, unless designed as part of an integrated system, can undermine the ability of purchasers to undertake strategic purchasing. Multiple funding flows are also associated with different levels of decision space and, in some cases, the possibility of cross-subsidization between purchasers and individuals (5, 14). The existence of multiple funding flows could aide or bring about improvements in health financing. However, previous studies have always focused on influence of a single funding flow or purchaser without considering the combined effects of multiple funds to health facilities (5, 15).

The study aimed to examine how healthcare providers respond to multiple funding flows and the implications of such flows for achieving the health systems goals of efficiency, quality, financial protection, equity, and resilience. It identified the different flows of funds to healthcare providers and characterized each funding flow by their inherent attributes. It also practically examined some theoretical provider responses to multiple funding flows and how the flows affect the health systems goals.

## Conceptual Framework

This study analyzed how multiple funding flows send signals that influence behavior of healthcare providers and the implications of these behaviors on health systems goals of efficiency, equity, and quality. Healthcare provider is used in this study to refer to organizations that provide healthcare services (e.g., hospitals), rather than individual healthcare workers working in these organizations or independently (e.g., doctors). A funding flow refers to any transfer of funds, in cash or kind, from a purchaser to a healthcare provider that is characterized by a distinct combination of arrangements.

**Figure 2:**
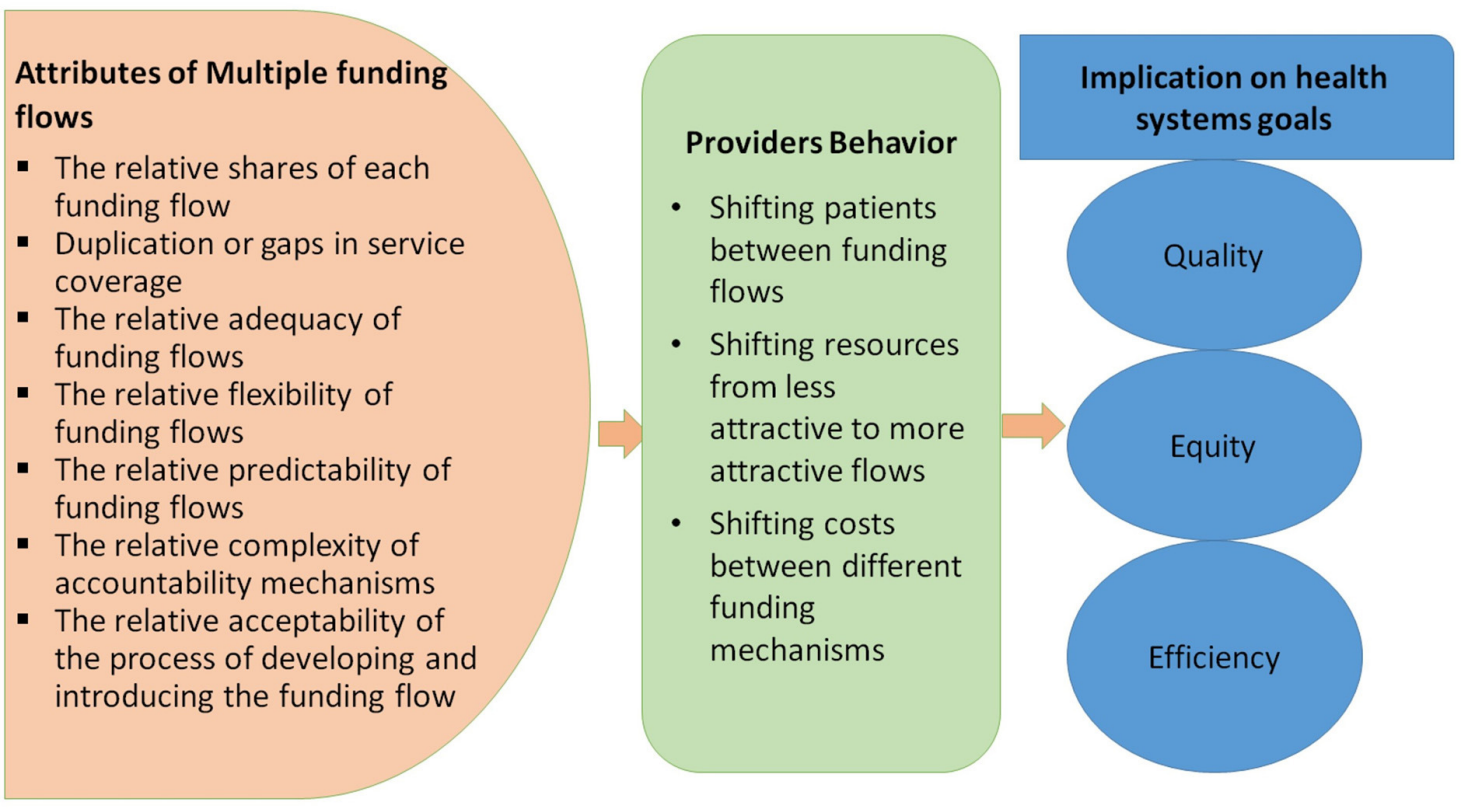
Conceptual framework for provider behavior to multiple funding flows in public health facilities.

Individual funding flows will have their own attributes/characteristics and incentives, which include: duplication or gaps in service coverage across multiple funding flows; contribution of each funding flow as a share of total; adequacy of funding flow to cover the costs of services purchased; flexibility that healthcare providers have over funding flow; accountability mechanisms; predictability; performance requirements; and inherent incentives generated by the provider payment mechanisms. The presence of multiple funding flows creates an additional layer of response defined by the relativeness of these attributes across funding flows. The interaction of these characteristics across funding flows could generate any of three behavioral responses among providers, namely: (i) shifting costs between different funding mechanisms; (ii) shifting patients between funding flows; and (iii) shifting resources from less attractive to more attractive flows (Figure 2).

Each of these provider responses occurs when healthcare providers value or prefer one funding flow over another due to a higher rate of financial return, or to other characteristics of the funding flow. It is also possible that there are positive consequences of multiple funding flows—for example, opportunities to cross-subsidize patients with lower financial capacity, enabling equity; or covering deficits in one funding flow through another, covering gaps in services, or shifting resources to more cost-effective services. The operational definitions are provided in Table 1.

**Table 1:** Operational definitions of provider behavior to multiple funding flows.

**Behavior**	**Definition**
Resource shifting	Resources (staff time, attention, beds, materials, equipment) moved to a more attractive funding flow
Patient shifting	Patients relocated from less profitable to more profitable funding flows/payment methods. This can also include situations where patients/services are shifted outside the facility, resulting in unnecessary admissions, treatments or charges
Cost shifting	Providers charge different rates to different purchasers for the same service. Providers charge more services to purchasers with higher payment rates or more attractive payment features than they do to other providers. One purchaser can be considered as overpaying while the other considered as underpaying (shifting the expected burden of costs). Cost shifting may also occur as over-billing to purchasers or individuals
Extra-billing	This means provision of additional services, not necessarily medically justified, to more generous purchasers
Service shifting/Patient transfer	A situation whereby a patient is transferred outside the facility for reasons that are not medically justified; horizontal referral (to another facility, e.g., private sector) or vertical referral (to a higher level facility)
Over-treatment	A provider decides to over-treat a patient to generate additional resources (over-prescription, unnecessary admission, DRG creep, etc.)
Patient selection	Providers give priority to patients with financially more attractive remuneration rates (patients with higher remuneration rates or patients that are less costly to be treated)

## Methods

### Study Sites and Design

It was a mixed-method cross-sectional study of selected healthcare providers and purchasers in Enugu state. The healthcare providers were the unit of analysis. Four public hospitals were purposively selected (2 tertiary and 2 secondary) on the bases that in addition to health insurance payments, they received funding from more than one healthcare financing mechanism. Out of the four public tertiary hospitals in Enugu state that received multiple flows, we selected the two biggest tertiary hospitals that were in the state and the secondary facilities that were affiliated to the two tertiary hospitals as their training outposts. Hence, we selected the University of Nigeria Teaching Hospital (UNTH) and Enugu State University Teaching Hospital (ESUTH). The secondary hospitals were Enugu State University Medical Center (ESUT Agbani) and Comprehensive Health Center, Obukpa (CHC Obukpa). UNTH and CHC Obukpa belong to the federal government, whilst ESUTH and ESUT are owned by the Enugu state government. The study was part of a multi-country study that was undertaken by the Responsive and Resilient Health Systems Consortium (RESYST), which had no impact on the data collection and findings from the study.

### Sampling

Key informants were purposively selected among hospital managers, administrators, and frontline health workers, as well as relevant officials from State Ministry of Health (SMoH), State Health Board (SHB), National Health Insurance Scheme (NHIS), and HMOs. The respondents were purposively selected based on the key consideration of people that will be able to provide the requisite information. Service users from each hospital were also purposively selected for focus group discussions (FGDs), to represent the range of funding flows in that hospital. A total of 66 key informant interviews (KII) and 8 FGDs were conducted. The FGDs comprised of 6–8 participants and were disaggregated by gender category (male and female). The service users were stratified by gender in the FGD to ensure that the opinions of the females were heard, otherwise the males will dominate the discussions as happens in the study context.

### Data Collection and Analysis

Primary data was collected through qualitative and quantitative methods. Key informant interviews (KIIs) and FGDs were conducted using pre-tested topic guides. Information on hospital characteristics and size of funding flows was collected using a structured checklist.

The interviews and FGDs were conducted by a team of trained researchers. All interviews were audio-recorded and lasted an average of 60 min. The FGDs were conducted in the local language (Igbo language) and translated into English. All audio recordings were transcribed verbatim and notes taken during the interviews were appended to the transcripts. The interviews were conducted between November and December 2017.

All transcripts were anonymised with pseudonyms. Deductive analysis of transcripts followed a rigorous process that started with familiarization of the transcripts to identify recurrent/common themes; generation of a provisional list of codes that were based on the research objectives, topic guide questions, and recurrent themes; testing of the provisional coding scheme; revision of the coding scheme and application to the rest of the transcripts. Coded data were sorted and relationships between participant categories and their perceptions/experiences explored. Coding was guided by including and comparing coded elements, leading to the identification of patterns and explanations.

Three different coding schemes were generated for the three categories of respondents namely, purchasers, service providers and service users. The final codes that were used in analysis are presented in Table 2.

**Table 2:** The coding scheme used in data analysis.

**Purchasers**	**Service providers**	**Service users**
Selection of health care providers	Sources of funding	Health service need and utilization in last 1–2 years
Provider payment methods and rates	Funding flows	Membership of health insurance scheme
Size of funding in past 3 years	Size of funding (how does it influence service delivery)	Non-membership of health insurance scheme
Contractual agreements with health providers	Duplication and gaps in service coverage of funding flows	
Relative adequacy of funding for services covered		Care experience in health facility
Relative flexibility of different funding flows		
Relative complexity of accountability mechanisms for different funding flows		Fairness in health service provision/delivery
Relative acceptability of process of development and introduction of funding sources and mechanisms		
Relative predictability of funding sources		Recommendations for improvement in equity, quality and efficiency
Conflicting incentives as a result of multiple funding flows		
Other experiences/benefits/challenges of having multiple sources of funding		
Shifting costs between different funding mechanisms Shifting patients between funding flows Shifting resources from less attractive to more attractive flows • Nature of … (type of …., how does it happen and to what extent?) • For whom/what … (patient groups/services) • Why does it occur (related attributes of funding flows) • Implications on health systems goals of quality, equity, and efficiency		

### Ethical Considerations

Ethical approval was obtained from the Health Research Ethics Committee of University of Nigeria Teaching Hospital Ituku-Ozalla, Enugu State, Nigeria. Written consent was obtained from each participant before the interview. Participants were provided with an information sheet that contained a brief description of the purpose of the study, their rights as participants and measures that will be taken by the research team to ensure confidentiality of information given.

## Findings

### Characteristics of Participants and Study Sites

Table 3 shows the category of key informants and FGD participants. In the KII, there were 66 respondents, of which 51.5% of them were females. Majority of the respondents (53.0%) were frontline health workers (Doctors, Nurses, Pharmacists, Laboratory scientists, CHEWs), 22.7% were purchasers and hospital managers/administrators were 13.6%. In the FGDs, there was an equal number of male and female participants. Thirty-two percent of the participants were petty trader/businessman, 28.8% were students while 11.5% were either retirees or pensioners.

**Table 3:** Characteristics of participants.

**Key informantsVariable**	**No of participants**	**Percentage**
**Gender**		
Male	32	48.5
Female	34	51.5
**Roles**		
Purchaser	15	22.7
Legislator	1	1.5
Hospital managers/administrators	6	3.96
Medical records/Accounts clerk/Medical Stores	9	13.6
Frontline health workers (Doctors, Nurses, Pharmacists, Laboratory scientists, CHEWs)	35	53.0
**Total**	66	100
**FGD participants (service users)**		
**Gender**		
Male	26	50
Female	26	50
**Source of funding**		
Direct OOP	34	65.4
NHIS (FSSHIP,TISHIP)	18	34.6
**Occupation**		
Petty trader/business man	17	32.7
Student	15	28.8
Retiree/pensioner	6	11.5
Civil/public servant	5	9.6
Artisans	2	3.8
Farmer	2	3.8
Un-employee	1	1.9
Other (Sales girl, cleaner, Security guard)	4	7.7
**Total**	**52**	**100**

*A summary of the characteristics of each hospital in terms of the number of outpatients, number of in-patients, and numbers of bed spaces (expected and observed) is shown in Table 4*.

**Table 4:** Characteristics of study facilities.

**Characteristics of study facility**	**UNTH**		**ESUTH**		**ESUT Agbani**		**CHC Obukpa**	
	**2016**	**2017**	**2016**	**2017**	**2016**	**2017**	**2016**	**2017**
Total number of Outpatient visits	164,089	137,787	27,531	30,031	2,879	2481	2,091	1,590
Total number of Inpatient admissions	7,399	5,957	8,094	8,084	431	590	284	250
Number of bed spaces that the hospital has	500	500	337	337	10	10	30	30
Number of beds (actual)	435	435	320	320	10	10	30	30

### Mapping of Funding Flows to Public Hospitals in Enugu

The data shows that funding flows to the public hospitals surveyed were multiple and varied by type of health facility (Secondary vs. Tertiary), ownership of health facility (Federal government vs. State government) and population served. Table 5 highlights respondents' reports of the various forms of funding to the hospitals. OOP and government budget were the only recurring forms of funding for all the public hospitals. The other common forms of funding were social health insurance and donations from development partners and voluntary agencies. Other sources of funding were PHI which was only available to tertiary hospitals and the drug-revolving fund which was only available to Federal government-owned hospitals.

**Table 5:** Variation in major forms of funding to public hospitals.

**Facility name**	**Facility type**	**Ownership**	**Population served**	**Major forms of funding**			
				**Government budget**	**Out of pocket payment**	**NHIS**	**Donations**
UNTH	Tertiary	Federal	General population				
ESUTH	Tertiary	State	General population				
ESUT Medical Center	Secondary	State	Staff and students				
CHC Obukpa	Secondary	Federal	Rural community				

*Key*.

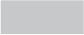
*Yes*.

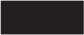
*No*.

The findings showed that the major sources of funding for both inpatient and outpatient services in both the state and federal government-owned health facilities, tare OOP, the NHIS and government budget. Donations are also used to fund some of the services to a lesser extent. The mechanism through which NHIS and government funds flowed from purchasers to providers varied for State-owned and Federal-owned public hospitals. NHIS payments from HMOs to State government health providers for capitation are first made into the centralized government account and then transferred to the hospitals. However, for fee-for-service payments from the NHIS, which is another funding flow, the funds are transferred directly to the hospitals by the HMOs after verifying the claims. Whereas, for Federal government-owned health providers, it is paid directly to hospital accounts. With respect to the o government budget, there were three purchasers for State-government hospitals namely, State Ministry of Health, Project Implementation Unit and State Ministry of Finance. Whereas, for Federal government-owned hospitals, the Federal Ministry of Health was the only government purchaser.

Each of the major funding flows to the four public hospitals is described in terms of services covered, target population, provider payment mechanisms and accountability mechanisms (Table 6).

**Table 6:** Characteristics of funding flows in terms of services purchased, target population, provider payment and accountability mechanisms.

**Funding source**	**Funding flow**	**Services purchased and target population**	**Provider payment mechanism**	**Accountability mechanism**
Government budget	Personnel (Salaries)	Staff salaries	Staff salaries are paid monthly	Electronic transfer of staff salaries in FG-owned hospitals Periodic financial audits of staff payroll in both FG-owned and SG-owned hospitals
	Recurrent budget	Direct subventions for overhead and other recurrent expenditure This is applicable for FG-owned hospitals	Monthly payments for overhead and other recurrent budgets	Electronic transfer, Documentation of income and expenditure, monthly reporting, monitoring visits by Ministry of Health
	Capital budget	Capital vote for infrastructure and equipment	Capital projects are implemented when needed, depending on funds available	Tendering receipts for capital expenditure; inspection of capital projects for quality and compliance to standard
	Free-MCH payments	State government funds free MCH which covers maternity and child health services for eligible mothers and children under 5 years of age	For free-MCH, periodic reimbursements are made on a case-by-case basis	Periodic financial audits
	Drug-revolving fund	Drugs that are purchased through direct out-of-pocket payments or cash transfers	One-off payment to FG-owned hospitals	Documentation of income and expenditure, periodic reporting, monitoring visits
Out of pocket payment		OOP can be used to purchase all services provided in public hospitals—consultations, laboratory tests, drugs, and other procedures. Also used to pay for utility bills and other consumables for service delivery	Cash payments direct from clients at the point of receiving care for services utilized (or yet to be utilized). Funds are transferred to Treasury Single Account and returned to hospitals	Automated electronic payment system tracks all payments made by clients Cash invoice to clients as evidence of payment. Monthly financial reporting Internal and external financial audits
NHIS	Capitation	Consultations, laboratory tests, drugs, and simple procedures listed in the NHIS benefit package. Also contributes to hospital revenue used for utility bills, infrastructure maintenance, and purchase of equipment FSSHIP covers federal government employees and beneficiaries. TISHIP covers students of registered tertiary institutions	Monthly capitation for primary level care. Capitation for FSSHIP is a fixed rate of ₦ 750/beneficiary and for TISHIP is ₦ 1,000/student	Authorization is required from HMOs for services not listed under capitation or FFS payments Periodic audits of hospital accounts by NHIS and HMOs. Periodic verification of payments made by HMOs' to the hospitals Technical committee approvals
	Fee for service	Secondary and tertiary level care as listed in the benefits package—surgeries, complex procedures, admissions	Monthly payments based on calculations. FFS rates vary depending on service type. Clients make 10% co-payment for FFS and drugs	
Donations		Cash or in-kind donations earmarked for specific services such as drugs and test kits for HIV, vaccines for immunization Overseas and local missions provide free surgical procedures. Philanthropists offset hospital bills of indigent clients	Donations are sometimes paid directly into the hospital account or given to the clients. Drugs and commodities for HIV treatment and care are given directly to the pharmacy unit of the HIV clinic	Similar to accountability mechanisms for government budget and OOP

### Characteristics of Major Funding Flows to Public Hospitals

The attributes or characteristics of each major funding flow is described in relation to other funding flows. Evidence from one of the tertiary hospitals shows that government funding for personnel cost contributes the largest share to overall hospital funds (63.1%). This is followed by OOP (17.9%), government funds for overhead (15.3%) and NHIS capitation (2.0%). Others are government funds for capital (1.4%) and NHIS-fee for service (0.3%). Details of other attributes of funding flows are presented in Table 7.

**Table 7:** Summary of relative attributes of funding flows to public hospitals.

**Attributes**	**Government funding**			**Out of pocket payment**	**NHIS**		**Donor funds**
	**Salaries (personnel)**	**Recurrent budget**	**Capital budget**		**Capitation**	**Fee for service**	
Relative share of funding	Highest share	Third highest	Fourth highest	Second highest	Fifth highest	Less share	Least share
Duplication or gaps in service coverage	No gaps or duplication	Does not cover highly specialized services	No gaps or duplication	Gaps—many people cannot afford the cost of highly specialized services	Gaps—NHIS drug formulary is restrictive		Duplication—donors run parallel programs as other funding sources
Relative adequacy of funds	Most adequate	Least adequate	Low adequacy—depends on availability of funds	Adequacy is low because services are subsidized	Capitation rate is inadequate but pooled capitation is moderately adequate	FFS rate and payments are highly inadequate	Moderately adequate for earmarked services
Relative flexibility of funding flows	Not at all flexible	Very flexible	Not at all flexible	***Varies***. Highly flexible in tertiary hospitals. Not flexible in secondary hospitals	Highly flexible and centrally pooled with other flexible sources	Highly flexible	Moderate flexibility
Relative predictability	Most predictable in terms of timing and amount	Highly unpredictable	Most unpredictable	Majority opinion is that it is highly predictable	Highly predictable in terms of amount Less predictable in terms of timing	FFS is less predictable in terms of timing and amount	Very irregular and has the least predictability
Relative complexity of accountability mechanisms	Less complex compared to OOP because personnel budget, which contributes the largest share, is earmarked			Most complex. Requires extra vigilance of accounting staff	Less complex than OOP but more complex than GF		Least complex. Funds are earmarked
Acceptability of process of developing and introducing funding sources	Less acceptable Decided by central government and lacking in fairness and accountability			More acceptable. Rates were decided by a committee	Least acceptable. Current design and rates were decided at the national level. Benefit package is not robust		Less acceptable Decision is made by donors.

### Providers' Behaviors to Attributes of Multiple Funding Flows

Table 8 shows that the relative attributes/characteristics of multiple funding flows to public hospitals send signals to providers that trigger responses such as resource shifting from less profitable/valuable to more profitable/valuable funding flows, patient shifting to more profitable or valuable funding flows, and cost shifting across funding flows to make up for inadequacy of funds. These provider responses/behaviors resulted in a differential quality of care for clients, inequities in access to health services, and wastage of resources. In some instances, providers' responses resulted in a better quality of care for clients and improved access to services that were not ordinarily available or clients could not have been able to afford otherwise.

**Table 8:** Evidence of provider behavior, related attributes, and implications for health systems goals.

**Types of provider behavior**	**Evidence of provider behavior**	**Related attribute/characteristic**	**Implications for health system goals**
RESOURCE SHIFTING(from less valuable to more valuable funding flows)	Assignment of designated doctors, nurses in outpatient department (OPD) and pharmacy for insured (NHIS) clients although the overall doctor/nurse-patient ratio in facility is low. Designated doctors are better qualified. This occurs because NHIS contributes significantly to hospital funds (size of funding). And the hospital needs to ensure continued patronage of NHIS clients, as well as to honor the MoU with NHIS-HMOs (State-owned tertiary hospital) “*Like the NHIS people are being given preference…in the out patients' unit. We have the doctors that are assigned to be seeing the NHIS patients when they come…despite the crowd or whatever. They have assigned doctors that see only them, and they also do it in other clinics. Even when they come with their children, you also give them attention”* (KII /R23)	Relative share of funding	NHIS clients get better quality of care than uninsured clients because waiting times are reduced
	Funds meant for drug revolving fund (DRF) for uninsured clients are used to purchase drugs for the NHIS pharmacy to prevent stock-outs that arise from delays in capitation payments in the Federal government-owned tertiary hospital. This results in depletion of DRF stock and delays in paying suppliers	Relative predictability of funding Relative flexibility	Depletion of the DRF funds for uninsured clients
	Health facility staff are shifted to philanthropy provided services (eye care, dental care, surgeries)	Gaps in service delivery	Improves access to specialized health services for the community
	Cardiothoracic unit is prioritized for resource mobilization (basic amenities, drugs and staff) during the annual free open-heart surgery programme provided by medical missions (VOOM foundation) to UNTH. This programme is valuable to the hospital because it fills a gap in service delivery “*What we are seeing in this current management is that interest is in open heart surgery, we know there are mission people in Diaspora coming to assist but the management attention has completely gone to that place to the detriment of every other aspect….” (*FP/KII/R05)	Gaps in service coverage associated with funding flows	Other health care services are under-resourced for the period resulting in differential quality of care
	In ESUTH, TB, and immunization clinics are under-resourced compared to other clinics because they do not generate any revenue for the hospital (services are provided free of charge) “*I will give you a typical example, immunization unit and the TB clinic are not well funded by the hospital the way they fund the SOP. The reason is that they don't see the money. Now, the quality of services rendered by TB and immunization cannot be compared to anything you run in clinical services because the funding doesn't come directly to them”* (FP/KII/R29)	Relative share of funding	Quality of services is poorer in these clinics (long waiting time & unconducive environment)
PATIENT SHIFTING(from less profitable to more profitable funding flows)	NHIS clients are made to pay user fees (the difference in fees) when drugs are prescribed outside of the NHIS-approved drug formulary. Purpose is to make up for inadequacy of NHIS billing as well as avoid delays in HMOs' authorization process. “*There are some drugs that are not in the list of NHIS approved for their enrolees, so if you have a case like that you have to go and buy the drugs by yourself and pay…At the moment what we actually do is to subtract the amount. For instance, for a brand of Ceftriaxone that is sold at *₦* 3,600, if the price [on NHIS drug list] is *₦* 600, we subtract the *₦* 600 and work out its 10% [co-payment] which is *₦* 60…So, the person is paying *₦* 3,060”* (FP/KII/R32)	Relative adequacy and predictability (time) of funding flows	Ensures that clients get the quality of services they require. Also has equity implications for insured clients who cannot afford the user fees
	Some NHIS clients are shifted from capitation to fee-for-service for expensive procedures that are not sufficiently covered by NHIS capitation payment	Relative adequacy of funding flows	Ensures that clients get the quality of services they require
	NHIS clients are made to pay OOP for services that are not covered by capitation due to communication gap between HMOs and health facility	Accountability	Implications for efficiency and equity (cost escalation)
	Free MCH—mothers are made to pay part of the fees to make up for unpredictability (time and amount) of reimbursements	Relative predictability	Implications for equity
	OOP clients are shifted from non-commercialized (public-funded) to commercialized (privately funded) laboratories in the hospital for two reasons: (i) the private laboratory offers wider range of investigations, is accessible at all times and has quicker turn-around time; (ii) the private laboratory generates more revenue for the hospital “*…There is a part of the hospital that runs their laboratory services and charges like a private place and the management knows they can get money directly from there so they do not fund the public typical labs so that these ones are not running and people will go to the private places.…where they pay more so that they can get what they want. Because government is not funding that one* (public labs) *it has made the services to go down. At a point it stopped running some tests”* (FP/KII/R10)	(i)Gaps in service coverage associated with funding flows (ii)Relative share of funding	Improves quality of care for those that can afford but creates inequities in access
	Different fees are charged to out-of-pocket paying clients for the same laboratory tests depending on whether they use the commercialized (privately-owned) laboratories or the non-commercialized (public-owned) laboratories in the hospital	Relative adequacy of funding flows	Improves quality of care for those that can afford but creates inequities in access
COST SHIFTING (different rates are charged to different funding flows for the same service)	NHIS is charged higher rates than out-of-pocket payment for the same laboratory investigations in UNTH The privately managed laboratory in UNTH charges higher fees than the public laboratories for the same laboratory tests. The private-owned laboratory operates like other for-profit private laboratories outside the hospital	Relative adequacy of funding flows	Inefficiency

### Positive Experiences of Having Multiple Funding Flows

Some respondents highlighted that having multiple sources of funding has been beneficial to the hospital in various ways. It increases the financial pool or internal revenue base of the hospital, which enables provision of wider range of services. Hence, client patronage is better. The capacity of the health facility to undertake capital projects from its internal revenue is also enhanced. Having multiple sources of funding also gives a sense of security to the health facility, in the sense that there would always be a back-up source of funding if one flow delays or defaults in payment.

“*It has a lot of benefits, when you have multiple fund, you see that you have sources of money that will help you to do what you want to do”* (FP/KII /R23)

“*It is a good thing that we have multiple sources; if this one is failing, you will lay hands on the other one, but if you have only one channel and it fails then you will be in trouble, so it is okay for us”* (FP/KII /R19)

“*Well, I will say that definitely having multiple source of funding has improved service delivery, because it has given so many options…definitely it is going to increase the financial pool. If you have a larger base, then it means that you can do more works”* (FP/KII/R25).

## Discussion

This paper provides new knowledge on how healthcare providers respond to multiple funding flows and the implications of such flows for achieving the health systems goals of equity, efficiency, and quality. It has identified the different flows of funds to healthcare providers and characterized each funding flow by ascertaining: the contribution of each funding flow to total resources; characterized a number of provider responses to multiple funding flows; and analyzed the likely impact of the overall funding mix on efficiency, quality, financial protection and equity in the healthcare services provided.

The findings showed that the different funding flows send signals to health providers that trigger responses such as resource shifting from less valuable to more valuable funding flows, patient shifting to more profitable or valuable funding flows, and cost shifting across funding flows to make up for inadequacy of funds. This also aligns with findings in other studies that health providers respond to signals sent by different funding flows (16, 17). Resources were also shifted to funding flows that providers find valuable because they address a gap in service delivery. Patients were shifted from less predictable to more predictable funding flows. They were also shifted from funding flows that had more complex accountability mechanisms to those that had less complex mechanisms. Providers charged different rates to different funding flows to make up for inadequacies in sources of funding.

The result also showed that although, government budget contributes the largest share of funding to public hospitals, over three-quarters of the fund is used for payment of staff salaries. This means that what is essentially available to the hospital for the provision of health service is small compared to OOP. This is in keeping with existing evidence which estimate that OOP contributes the most to hospital revenue for actual service delivery (18, 19).

The mechanisms through which funds flowed from purchasers to providers varied by source of funding, services purchased and facility type as was found in this study. Government budget for staff salaries in State-owned hospitals was paid through the Ministry of Finance while that for Federal government-owned hospitals are paid through the Ministry of Health. Similarly, funds for capital projects in state-owned hospitals were managed by the State Project Implementation Unit, unlike in Federal government-owned hospitals. Concerning variations based on the type of service purchased by the same source, funding for HIV treatment and care was provided through implementing partners while funding for other programmes was provided through State or LGA health authority. Generally, majority of the funds did not flow directly from purchasers to providers, and for a single hospital, there were multiple actors from different sectors involved in the flow of funds from purchasers to service providers.

An implication of the findings is that multiple funding flows to public hospitals is beneficial as well as constraining to health providers. It provides collateral funding pathways that improve overall flexibility, predictability and adequacy of funding to the hospital. Hospital revenue is increased and more resources (funds) are available for the provision of a wider range of services. As more services are provided with better quality, client patronage improves. These findings corroborate a recent study which found that multiple funding flows reduced the interruption of service delivery as a result of lack of equipment or medical supplies (20) and increased providers' funds and patient flow to the facilities (21). Collateral funding pathways have also been reported to contribute to strengthening organizational resilience (22). The capacity of health facilities to undertake capital projects from their revenue is also enhanced by multiple funding flows.

The characteristics/attributes of individual funding flows are influenced by the presence of multiple sources of funding and this study highlights some of the challenges of multiple funding flows to public hospitals. Providers perceive the size/share, adequacy, predictability, flexibility and complexity in accountability of a funding flow in relation to other sources of funding. In addition to the fact that multiple funding flows result in duplication of services and complicates financial management and accountability, the interaction of attributes of different funding flows sends signals that fuel discriminatory behavior among providers.

Providers' perception of relative adequacy of funding flows shaped their behaviors in health service delivery in this study. For instance, because NHIS capitation and FFS were considered to be inadequate and the benefits package limited, providers shifted patients to direct OOP to maintain the continuation of care. Although this was done with good intentions to ensure that clients got the best quality of care that was available, it could potentially result in inequity in access because those who cannot afford the additional cost of care are denied treatment as has been reported elsewhere (23, 24). Furthermore, providers were found to prioritize resources (personnel, medicines, space, electricity, and water) for those services whose funding flows generated additional revenue for the hospital. The effect of this resource prioritization is that clients whose funding flows were not considered profitable received services that were under-resourced and of poorer quality. A similar finding was reported in Kenya where it was found that resources were preferentially allocated to National Health Insurance Fund beneficiaries by hospitals (25).

Predictability of funding flows was reported in this study by the providers in terms of completeness and timeliness of payments for services delivered (or to be delivered) to clients. Funding flows whose payments were closely aligned with expectations of providers were considered relatively more predictable than others. The relative predictability of these funding flows influenced how providers responded with service delivery to clients. It enabled resource shifting from less predictable sources to more predictable sources. Providers prioritized resource allocation to NHIS over OOP because the later was considered less predictable, being always affected by frequent strike action of health professionals. In one of the hospitals that receive government funding for free MCH programme, patient shifting was also reported because reimbursements are oftentimes delayed and/or incomplete. Suitability for free MCH was sometimes ignored and eligible women and children were made to pay user fees for maternal and child health services. It was reported that unpredictability of a health care programme funds resulted in drug stock-outs in public facilities which compelled providers to introduce informal payments among service users for services that should be provided free of charge in Nigeria (26). This behavior facilitates and widens the inequity gap that already exists in access to maternal and child health services in Nigeria because of the potential higher financing burden on the poor and vulnerable groups. It could potentially reverse the gains in maternal and child health outcomes, particularly those associated with increased utilization of formal health facilities. The equity of a health financing system does not only depend on how pro-poor the distribution of its benefits is but also how the financing burden is shared (24).

Accountability mechanisms reportedly varied across the different funding flows and some funding flows were considered to have more complex mechanisms for accountability than others. Complexity was defined by reporting requirements (content and frequency), processes of authorization, duplications in accounting processes and potential for diversion of funds under funding flows. The perceived complexity of accountability mechanisms sent signals to providers that resulted in patient shifting. The process of authorization from HMOs to providers for delivering services that are outside of the NHIS list was perceived to be highly bureaucratic and prolonged. In order to circumvent this process and reduce delays in service delivery, providers shifted clients from NHIS capitation to fee for service through direct cash payments. Although this behavior had positive implications for quality of care, it could create inequities in access since it preferentially benefits insured clients who can afford to make direct cash payments. In India, it was also found that delay in reimbursements from the National Health Insurance Scheme was also reported, which forced providers to reject enrollees and subsequently deregister themselves from the scheme (27).

The findings concerning the flexibility of funding flows showed that with the exclusion of earmarked government and donor funds, other funding sources had comparative flexibility and were pooled and managed from a central pool. This attribute generated varied behavioral responses from providers. On the one hand, it enabled resource prioritization by providers to more profitable funding flows. Hence, creating differential quality of care. On the other hand, it enabled redistribution of funds and resources across funding flows and this improved fairness in quality of care provided for all groups of clients.

It was found that the fact that the processes of development and introduction of funding sources were decided by authorities other than health providers was unacceptable to the providers. Provider payment rates did not cover the cost of services because NHIS capitation and fee for service rates were decided at the national level while user fees for OOP in public hospitals were handed down to providers without their inputs. In order to cope with the consequent inadequacy of funding flows, providers charged different rates to “vulnerable” funding flows for the same services (28). Specifically, in one of the hospitals, insured clients paid higher FFS rates for some investigations than uninsured clients, and laboratory fees were higher in privately managed laboratories than in the public laboratories. The non-inclusion of health providers in rates-fixing has been reported as detrimental to the quality of care (19). The discriminatory behavior of providers which is elicited has negative implications for efficiency when clients are overcharged for services (28). As posited by the World bank, overall efficiency in the health system can be increased by reforming procedures for purchasing services (29).

The main limitation of this study is that we relied on participants' reports of their perceptions of provider responses to attributes of multiple funding flows. Also, the study employed a non-probability sampling approach (purposive sampling method) in selecting both the study sites and participants which may have introduced bias to the overall findings. Furthermore, although we elicited information about the fund flow system in different health care facilities, we did not explore the financial control mechanisms, which is equally important for service delivery in low and middle income countries like Nigeria. The exploration of financial control mechanisms for different funding flows should be the subject of future studies.

In conclusion, multiple funding flows to public hospitals are beneficial as well as constraining to health providers. It is important that such multiple flows are better harnessed and channeled for improving health system financing and overall health system strengthening. These consequences of multiple funding flows to public hospitals affect health systems goals of equity, efficiency and quality of care. In the most part, they enabled inequity in access to health services and differential quality of care for different groups of clients.

## Data Availability Statement

The datasets generated for this study are available on request to the corresponding author.

## Ethics Statement

The studies involving human participants were reviewed and approved by University of Nigeria Teaching Hospital Ethics Committee. The patients/participants provided their written informed consent to participate in this study.

## Author Contributions

OO participated in the conceptualization of the study. OO and NE were involved in the development of the data collection tools. OO, NE, CM, UE, and IA participated in finalizing the data collection tools, data collection, and analysis. The manuscript was drafted by OO but all the co-authors were involved in its revision and finalization.

### Conflict of Interest

The authors declare that the research was conducted in the absence of any commercial or financial relationships that could be construed as a potential conflict of interest.
